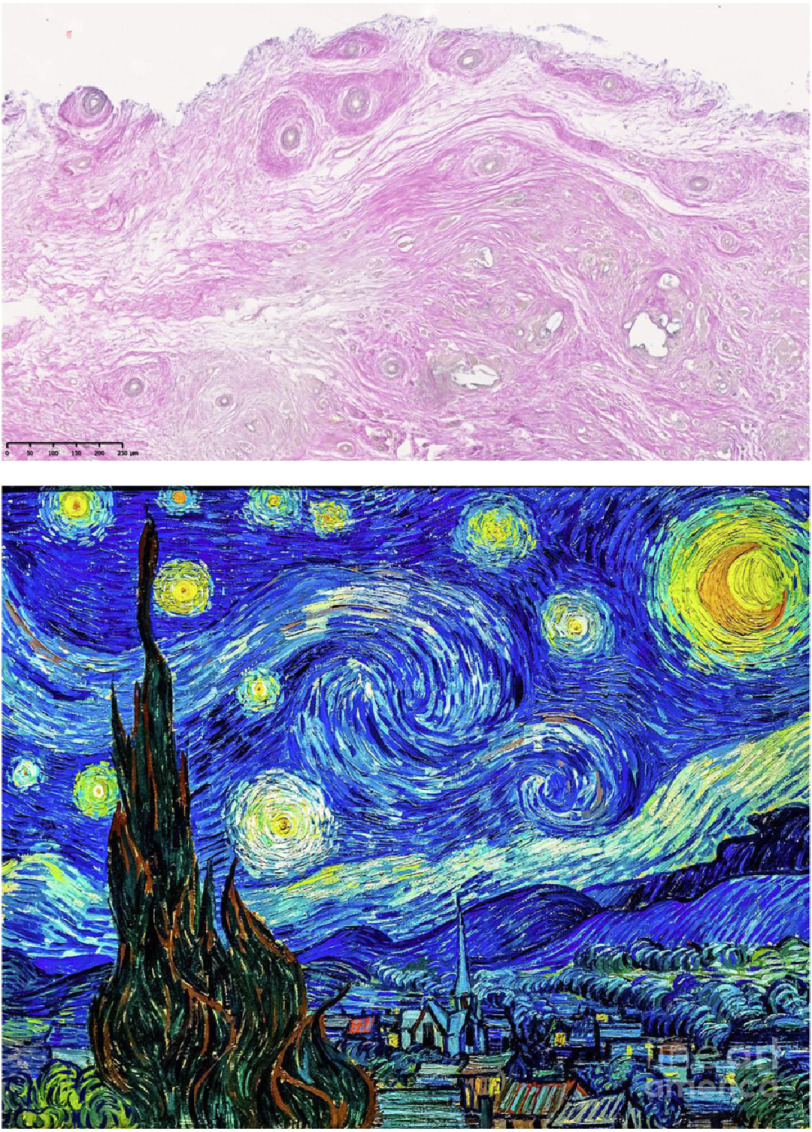# Starry Night by Van Gogh and morphogenesis of a tissue engineered heart valve

**DOI:** 10.21542/gcsp.2021.30

**Published:** 2021-12-31

**Authors:** Najma Latif, Yuan-Tsan Tseng, Magdi H Yacoub

**Affiliations:** 1 The Magdi Yacoub Institute, Heart Science Centre, Harefield Hospital, Hill End Road, Harefield, Middlesex UB9 6JH

A vivid example of art and science intertwining and reflecting across generations.

A clear demonstration of beauty and motion in biological iterations.

Angiogenesis and stars, the bringers of light and elegance to a dark sky.

Interspersing/intermingling collagen, moving clouds in the wind, not to deny

Sheets of collagen, a stable platform over the City of the Valve providing protection.